# Plasmid-encoded lactose metabolism and mobilized colistin resistance (*mcr-9*) genes in *Salmonella enterica* serovars isolated from dairy facilities in the 1980s

**DOI:** 10.1099/mgen.0.001149

**Published:** 2023-11-30

**Authors:** Carsten Kröger, Nicole A. Lerminiaux, Anna S. Ershova, Keith D. MacKenzie, Morgan W. Kirzinger, Erwin Märtlbauer, Benjamin J. Perry, Andrew D. S. Cameron, Kristina Schauer

**Affiliations:** ^1^​ Department of Microbiology, School of Genetics and Microbiology, Moyne Institute of Preventive Medicine, Trinity College Dublin, Dublin 2, Ireland; ^2^​ Department of Biology, University of Regina, Regina, Saskatchewan, S4S 0A2, Canada; ^3^​ Institute for Microbial Systems and Society, Faculty of Science, University of Regina, Regina, Saskatchewan, S4S 0A2, Canada; ^4^​ Department of Veterinary Sciences, Faculty of Veterinary Medicine, Ludwig-Maximilians-University Munich, Oberschleißheim, 85764, Germany; ^†^​ Present address: National Research Council Canada, Saskatoon, Saskatchewan, S7N 0W9, Canada; ^‡^​ Present address: AgResearch, 176 Puddle Alley, Mosgiel 9092, New Zealand

**Keywords:** colistin resistance, comparative genomics, *lac *genes, lactose, *mcr*-9, *Salmonella enterica*

## Abstract

Horizontal gene transfer by plasmids can confer metabolic capabilities that expand a host cell’s niche. Yet, it is less understood whether the coalescence of specialized catabolic functions, antibiotic resistances and metal resistances on plasmids provides synergistic benefits. In this study, we report whole-genome assembly and phenotypic analysis of five *Salmonella enterica* strains isolated in the 1980s from milk powder in Munich, Germany. All strains exhibited the unusual phenotype of lactose-fermentation and encoded either of two variants of the *lac* operon. Surprisingly, all strains encoded the mobilized colistin resistance gene 9 (*mcr-9*), long before the first report of this gene in the literature. In two cases, the *mcr-9* gene and the *lac* locus were linked within a large gene island that formed an IncHI2A-type plasmid in one strain but was chromosomally integrated in the other strain. In two other strains, the *mcr-9* gene was found on a large IncHI1B/IncP-type plasmid, whereas the *lac* locus was encoded on a separate chromosomally integrated plasmidic island. The *mcr-9* sequences were identical and genomic contexts could not explain the wide range of colistin resistances exhibited by the *Salmonella* strains. Nucleotide variants did explain phenotypic differences in motility and exopolysaccharide production. The observed linkage of *mcr-9* to lactose metabolism, an array of heavy-metal detoxification systems, and other antibiotic resistance genes may reflect a coalescence of specialized phenotypes that improve the spread of colistin resistance in dairy facilities, much earlier than previously suspected.

## Data Summary

The raw reads used for the genome-sequence assemblies were deposited in the Sequence Read Archive (SRA) under accession numbers (SRR23073758, SRR23073759, SRR24093640, SRR24093641, SRR24094119, SRR24094120, SRR24097258, SRR24097259, SRR24097305, SRR24097306) and BioProject numbers (PRJNA904948, PRJNA923601, PRJNA952980, PRJNA952981, PRJNA952982).

**Impact Statement**

*
Salmonella enterica* is a common food spoilage organism that is typically lactose-negative; however, lactose-positive *Salmonella* are sometimes isolated from dairy products. The evolution of lactose-positive *Salmonella* is therefore of interest for the dairy industry because the phenotype is used routinely for differentiation between *Salmonella* and other *Enterobacteriaceae* in food. We sequenced the genomes of all five lactose-positive *S. enterica* strains isolated in the 1980s from contaminated milk powder to discover that the strains contained two variants of the *lac* genes required for lactose metabolism. Close inspection of the genomes also revealed plasmid-encoded mobile colistin resistance genes (*mcr-9*), revealing that *mcr* genes have been circulating in *Salmonella* on plasmids decades earlier than previously reported. The genomes of the five strains also possess a large array of metal detoxification and antibiotic resistance genes, reflecting the extensive use of heavy metals and antibiotics in farm animal and dairy industries in the 1980s. Our study highlights the linkage of metabolic, antibiotic resistance and metal detoxification systems and demonstrates the value of revisiting historical strain collections with contemporary genome-sequencing technology to improve our understanding of bacterial evolution in food systems.

## Introduction

Bacteria from the pre-genomic era are kept in laboratories around the world without ever being revived for research. Today, the relative ease of bacterial genome sequencing allows scientists to revisit and sequence genomes of strains in historical collections to better understand bacterial evolution and environmental adaptation. Characterizing bacterial genomes of historical samples has improved our understanding of historical outbreaks and even pandemics. For example, sequencing genomes of *Vibrio cholerae* NCTC 30, which was isolated in 1916 from a British soldier, provided new insights into pathogen epidemiology and evolution [1], as did sequencing the genomes of ancient *Yersinia pestis* strains that were the origin of the fourteenth century plague pandemic [2].

Here, we revisited the culture collection at the Institute of Hygiene and Technology of Milk at Ludwig-Maximilians-University of Munich, Germany to investigate five unusual lactose-fermenting *Salmonella enterica* strains that were deposited in the 1980s. The five strains were isolated during routine testing for bacterial contamination of powdered milk obtained from dairy production companies in Northern and Southern Germany.

*Salmonella enterica* is one of the most frequent and problematic bacterial contaminants of food. *S. enterica *’s inability to metabolise lactose provides a convenient culture-based method to discriminate *Salmonella* from other *Enterobacteriaceae* such as *Escherichia* and *Shigella* when screening dairy and other food products for bacterial contamination. However, cases of lactose-fermenting *Salmonella* bacteria have been reported as early as 1907 (then called ‘*Bacillus typhosus*’) [3], and acquisition of the *lac* operon by *S. enterica* has been described [4]. *S. enterica* subspecies *diarizonae* and to a lesser extent subspecies *indica* more commonly demonstrate lactose metabolic capabilities [5]. Lactose-fermenting *S. enterica* have caused outbreaks in calves [6] and are anticipated to elevate the risk of contaminating milk-producing facilities; thus, finding the genetic determinants of lactose metabolism in *S. enterica* is important for food safety and animal welfare.

The wide use of antibiotics and metals in animal feed for growth-stimulating and prophylactic benefits is implicated as a major driver of antibiotic and heavy-metal resistance. Heavy metals have long been used as supplements in livestock feed to promote animal growth and to increase feed efficiency, which has increased since the EU ban of the use of antibiotics as growth promoters in animal feed in 2006 [7]. We used whole-genome sequencing to characterize lactose-positive *Salmonella* isolated from powdered milk, specifically examining the genetic bases of antibiotic resistance and virulence phenotypes relevant to human and animal health. Long-read DNA sequencing resolved plasmids and gene islands containing mobile colistin resistance gene *mcr-9* linked to heavy metal and other antibiotic resistances, demonstrating the value of revisiting the genomes of historical strain collections to identify risk factors and track bacterial evolution in farm and food industries.

## Methods

### Isolation and identification of lactose fermenting strains from powdered milk products

The five strains were isolated in the early 1980s from powdered milk products from different dairy companies in Northern and Southern Germany. The likeliest years of isolation are 1980 or 1981, when the strains appear in an internal report from 1981 of a project at LMU, which started late 1979. The exact dates of isolation have not been retained. To detect *Salmonella* spp., the international standard method (ISO/TC 34/SC 9) was carried out, which specifies a horizontal method for detection, enumeration and serotyping of *Salmonella* spp. in the food production chain [8, 9]. This method included pre-enrichment of food samples in a non-selective broth medium (buffered peptone water), enrichment in two different selective broth media (Mueller–Kauffmann tetrathionate and Rappaport Vassiliadis broths), isolation of presumptive *Salmonella* colonies on differential media plates Xylose Lysine Deoxycholate (XLD) and Mannitol Lysine Brilliant Green Crystal Violet (MLCB), biochemical screening (EnterotubeTM II for identification of *Enterobacteriaceae *) and serological confirmation [carried out by the National Reference Laboratory for *Salmonella* on the German Federal Institute for Risk Assessment (BfR, Berlin, Germany)].

### Phenotypic testing of lactose utilization

A colourimetric assay was used to assess the ability of bacteria to use lactose, glucose or *myo*-inositol as a sole carbon source [10]. Bacterial strains were grown from single colonies overnight at 37 °C and 220 r.p.m. in 5 ml lysogeny broth medium [Lennox (l-), 10 g/l tryptone, 5 g/l yeast extract, 5 g/l NaCl, adjusted to pH 7.5]. In total, 1 ml of cells was pelleted by centrifugation and washed twice in 1 ml PBS. The colourimetric assays were carried out in 96-well plates. Cells were adjusted to an OD_600 nm_ of 0.3 in 90 µl M9 medium supplemented with 2 mM MgSO_4_, 0.1 mM CaCl_2_, 0.03 % (w/v) pluronic F68 (Kolliphor, Sigma K4894), 0.02 % (w/v) gellan gum (Phytagel Plant, Sigma P8169), and 0.01 % (w/v) tetrazolium violet, mixed with 10 µl of the carbon source (stock concentration 0.05 M, *myo*-inositol, d-glucose or l-lactose) and grown for 48 h at 37 °C with agitation [10]. After 24 h and 48 h, the 96-well plate was measured at OD_620 nm_ in a plate reader (Tecan, Männedorf, Switzerland). For each strain and substrate combination, experiments were repeated with 13 independent biological replicates. As controls, *Cronobacter sakazakii* ES5 (lactose-positive [11]) and *S*. Typhimurium 4/74 were used (lactose-negative [12, 13]).

### Isolation of genomic DNA for whole-genome sequencing

Bacteria were grown overnight in 5 ml lysogeny broth medium [Luria–Bertani (LB-), 10 g l^−1^ tryptone, 5 g l^−1^ yeast extract, 10 g l^−1^ NaCl, adjusted to pH 7.5] medium, and 1.5 ml were pelleted by centrifugation. The supernatant was discarded, and the pellet was resuspended in 400 µl lysis buffer (100 mM Tris pH 8.0, 5 mM EDTA, 200 mM NaCl). After the addition of 100 µl lysozyme (10 mg ml^−1^ in lysis buffer), the cell suspension was incubated on ice for 15 min. Then, 10 µl sodium dodecyl sulphate (SDS, 10 %, w/v) and 5 µl proteinase K (10 mg ml^−1^) were added and the cell suspension was incubated overnight at 55 °C in a hybridization oven. The next morning, DNA was precipitated by the addition of 500 µl isopropanol and the precipitated DNA was transferred using a yellow pipette tip to a new 1.5 ml Eppendorf tube. The DNA was washed once with 96 % ethanol, once with 70 % ethanol and pellet air-dried. The DNA was finally resuspended in 500 µl TE buffer (pH 7.4) containing 10 µg RNase, and the concentration measured on a Nanodrop spectrophotometer.

### Genome sequencing

Short-read library preparation was done with the NEBNext Ultra DNA library kit for Illumina. Illumina libraries were sequenced on the MiSeq platform using Reagent Kit v3 (600-cycle, Illumina), producing 300 bp paired-end reads. For long-read sequencing of MHI916 and MHI966, the DNA was sheared with g-TUBEs to obtain 8 kb fragments following the manufacturer’s protocol (Covaris), while DNA from MHI917, MHI949 and MHI951 were not subjected to shearing step prior to library preparation. Long-read library preparation was done with the SQK-LSK109 ligation sequencing kit and the EXP-NBD103 native barcoding expansion kit (Oxford Nanopore Technologies). Oxford Nanopore Technologies libraries were sequenced on a MinION R9.4.1 flow cell and reads were base-called with the HAC model with Guppy v4.2.2 (MHI916, MHI966) or with the SUP model with Guppy v6.1.7 (MHI917, MHI949, MHI951). Illumina read depth was between 10-fold to 40-fold, and Nanopore read depth was 65-fold for MHI916, 32-fold for MHI917, 30-fold for MHI949, 83-fold for MHI951 and 20-fold for MHI966. The values listed represent average read depth of coverage across the entire assembly.

### Sequence assemblies

For Illumina reads, we used Trimmomatic v0.35 [14] to remove adapter sequences and eliminated reads with an average Qscore <30 across a 4 bp sliding window. For Nanopore reads, we used Porechop v.0.2.3 (https://github.com/rrwick/Porechop) to remove barcodes and adaptors, and Filtlong v0.2.0 (https://github.com/rrwick/Filtlong) to eliminate reads with a mean quality <90 and a read length <1 kb. For isolates MHI916, MHI917 and MHI951, we used Trycycler v0.4.1 [15] to generate a consensus long-read assembly from 50-fold read sets assembled by Flye v2.8.1 [16], Raven v1.3.0 [17], Canu v2.1.1 [18] and miniasm/Minipolish v0.3/v0.1.2 [19]. Isolates MHI966 and MHI949 had lower long-read coverage and therefore were not ideal for input into Trycycler, so we used Quast v0.5.2 [20] to evaluate competing assemblies for contiguity. We used Flye v2.8.1 for MHI966 and we used quickmerge v0.3 [21, 22] to merge assemblies of Canu v2.1.1 and Unicycler v0.4.7 [23] for MHI949. Assemblies were polished with Medaka v1.2.1 (https://github.com/nanoporetech/medaka) and Pilon v1.23 [24]. Mapped reads were visualized in Tablet [25] with BAM files generated by Minimap2 v2.17 [26] and Samtools v1.9 [27]. Genomes were annotated using Prokka v1.14 [28] and PGAP v5.0 [29]. Plasmids were identified based on forming circular contigs during assembly and presence of plasmid markers (replicon, relaxases, mating pair formation protein, *oriT* sequence) evaluated by MOB-suite [30]. Circularization of contigs is performed as part of the Flye and Unicycler assemblers and the Trycycler consensus pipeline.

### Genome and sequence comparisons and variant calling

Pairwise genome comparison were carried out using Mauve 2.4.0 [31] and multiple sequence alignments (MSAs) using Clustal Omega with default settings [32]. MSAs were subsequently visualised with JalView [33]. Variant calling was performed with Snippy version 4.6.0 [34].

### Genomic visualization

Genomic island visualizations were created with BRIG [35] and Easyfig [36]. For isolates that only had Illumina reads, we used Bowtie2 v2.4.2 [37] to generate SAM files for coverage mapping in BRIG. The *lac* island boundaries were identified by sequence alignment to model *Salmonella enterica* subsp. *enterica* Typhimurium SL1344 (NC_016810.1 [38]). Genomes with *lac* islands related to MHI966 (NC_010870.1, CP021463.1, CP042494.1, CP051271.1, CP021749.1) and MHI916 (JZTJ00000000.1, AHUY01000000.1, CP024168.1, CP028974.1, LR590464.1, CP049021.1) were selected from top blastn hits for four genera [39] and from related work [4].

### Phylogenetic analysis

A total of 544 sequences containing three genes of the *lac* operon (*lacI*, *lacZ* and *lacY*; excluding *lacA*), including multiple species were retrieved from GenBank by comparing the nucleotides from position 792 611 to 798 231 from MHI916 containing *lacI*, *lacY* and *lacZ* including their intergenic regions to the non-redundant nucleotide database (June 2021) using blast v.2.9.0–2 (e-value <0.01) [39]. Hits with coverage >90 % were taken for clustering. The *lac* operons were clustered by nucleotide sequence identity, using CD-HIT v.4.7 ‘-c 0.98 -aS 0.9’ [40]. Operons that possessed >98 % nucleotide sequence identity were considered as members of the same sequence cluster. Representatives of each cluster and five *lac* operons of analysed *S. enterica* genomes were aligned by muscle v3.8.31. A maximum-likelihood phylogenetic tree was generated using PhyML v.3.3.3 : 3.3.20170530+dfsg-2, 100 bootstraps were used [41] and visualized in iTOL [42].

### Antibiotic susceptibility tests

Antibiotic susceptibility of the bacterial isolates was performed by a disc diffusion assay (Oxoid, ThermoFisher Scientific) according to guidelines by the European Committee on Antimicrobial Susceptibility Testing (EUCAST [43]): Penicillin G (PenG, 10 µg), oxacillin (OX, 5 µg), cephalexin (CL, 30 µg), gentamicin (CN, 10 µg), trimethoprim/sulfamethoxazole (SXT, 25 µg), and ciprofloxacin (CIP, 5 µg). The MIC for colistin was determined by the broth micro-dilution (BMD) method and by a concentration gradient diffusion test (MIC test strips; Liofilchem srl, Italy) according to EUCAST and to the manufacturer’s instructions, respectively, with one variation: a MIC test strip of the following range of concentrations for colistin was included (256–0.016 µg ml^−1^). Colistin concentrations for BMD ranged between 0.0625 and 32 µg ml^−1^. Determination of the MIC of colistin with MIC test strips was carried out on Mueller–Hinton agar plates [0.75 % (w/v) agar]. In all cases, the bacterial cultures were incubated at 37 °C for 18–24 h.

### Colistin killing assay

The bacterial killing assay was essentially carried out as previously described [44, 45]. *Salmonella* strains were grown at 37 °C with shaking in Mueller–Hinton broth overnight. Cells from the overnight culture were diluted 1 : 100 in fresh Mueller–Hinton broth and incubated at 37 °C with vigorous shaking until they reached an optical density at 600 nm (OD_600 nm_) of 0.6 to 0.7. Bacterial cultures were then mixed with colistin at concentrations of 0 (mock, PBS-treated), 0.5, 1, 2, 2.5 or 5 µg ml^−1^ and incubated at 37 °C for 1 h. After the 1 h incubation, the samples were serially diluted in PBS and plated onto LB agar plates to determine the number of c.f.u. The % survival was calculated as follows: [c.f.u. of colistin-treated culture/c.f.u. of mock (PBS-treated) culture] × 100.

### Growth on milk agar

Whey-based formula milk agar was used in capsule production assays. To prepare milk agar, 3.6 g of agar and 0.4 g of ammonium sulphate were dissolved in 40 ml distilled water. After autoclaving, the ammonium sulphate-agar was mixed with 200 ml of sterile, liquid infant formula preheated to 55 °C (Aptamil Pronutra Pre, ready-to-drink formula from birth, Nutricia Milupa GmbH, Frankfurt am Main, Germany) and then plates were poured. The *Salmonella* strains were grown overnight on LB agar at 37 °C and a single colony was streaked out on milk agar and incubated overnight at 37 °C and at least 4 more days at room temperature.

### Motility assays

Motility assays were performed using *Salmonella* cells grown overnight on XLD agar plates. The cells were directly taken from the plate, washed once, and resuspended in PBS and set to an optical density at 600 nm (OD_600 nm_) of 2. Three plates with l-agar, l-agar with 7.5 g l^−1^ lactose and XLDmod (3 g l^−1^ yeast extract, 5 g l^−1^ NaCl, 7.5 gl^−1^ lactose monohydrate, pH 7.4±0.2) containing 0.3 % (w/v) agar were stab inoculated with 1 µl of *Salmonella* suspension and incubated upright at 37 °C. Motility was quantified by measuring the diameter of the motility zone.

## Results

### Lactose metabolizing *Salmonella enterica* from dairy products

**Fig. 1.**
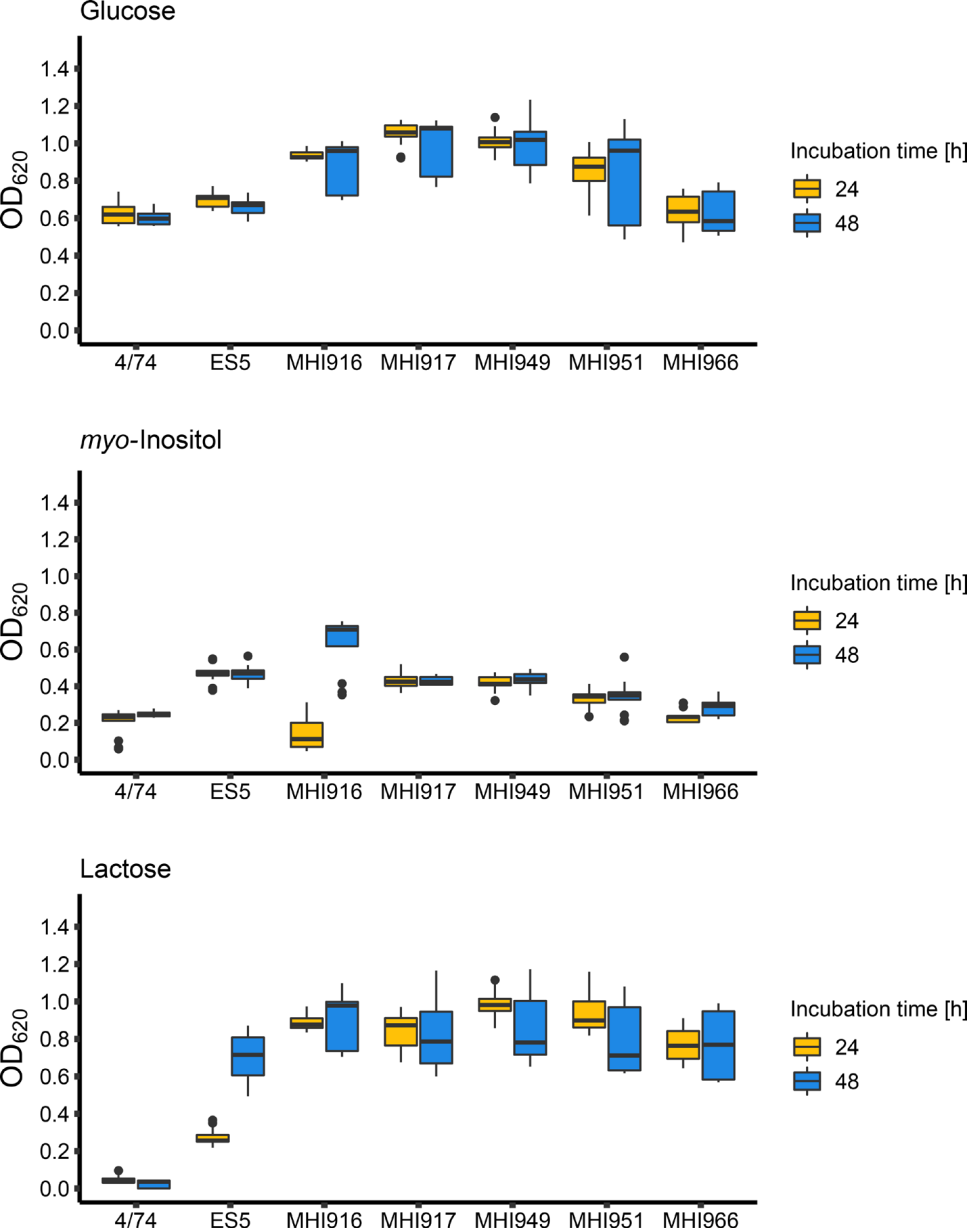
Phenotypic, colourimetric assay to measure the ability to metabolize carbon sources [glucose (top panel), *myo*-inositol (middle panel), lactose (bottom panel)] at 37 °C. Bacteria were grown in modified M9 minimal medium supplemented with tetrazolium violet and the indicated C-source. The optical density was measured after 24 (yellow) and 48 (blue) hours of incubation (*n*=12). *S*. Typhimurium 4/74 is labelled as 4/74, *Cronobacter sakazakii* ES5 is labelled as ES5.

During routine dairy product monitoring by the Institute of Hygiene and Technology of Milk at Ludwig-Maximilians-University of Munich (LMU), Germany, in the early 1980s (see Methods), five lactose-positive *Salmonella* strains (named MHI916, MHI917, MHI949, MHI951 and MHI966) were isolated from powdered milk products from three different dairy companies located in Northern and Southern Germany and stored in the local strain collection at −80 °C (Table 1). Because lactose metabolism is a rare phenotype in *S. enterica *, we sought to elucidate the genetic compositions of these strains. Fortunately, the strains were found to grow well on l-agar at 37 °C after more than 30 years of storage at −80 °C. To confirm that the isolated strains metabolize lactose as a sole carbon source, a colourimetric assay to track the reduction and concomitant change of colour of tetrazolium violet as a final electron acceptor during respiration was conducted ([10], Fig. 1). All five *Salmonella* MHI strains were able to metabolise glucose and *myo*-inositol positive controls (Fig. 1). Strain MHI916 showed slightly slower utilization of *myo-*inositol, which could be explained by the long lag phase of some *Salmonella* strains when grown with *myo*-inositol as a sole carbon source [10, 46, 47]. *Cronobacter sakazakii* ES5 was used as a lactose-positive control and *Salmonella enterica* serovar Typhimurium (*S*. Typhimurium) 4/74 served as a lactose-negative control (Fig. 1). All five *Salmonella* MHI strains were able to metabolize lactose as the sole carbon source, confirming the lactose-positive phenotype (Fig. 1).

**Table 1.** Overview of lactose-positive *Salmonella enterica* strains isolated from dairy products in the 1980s. Serovar and MLST predicted by SISTR

Species	Strain	Serovar	MLST	Origin
* Salmonella enterica *	MHI916	Ohio	329	Bavaria, Germany, Dairy Company no. 1
* Salmonella enterica *	MHI917	Ohio	329	Bavaria, Germany, Dairy Company no. 1
* Salmonella enterica *	MHI949	Agona	13	Lower Saxony, Germany, Company no. 2
* Salmonella enterica *	MHI951	Agona	13	Bavaria, Germany, Dairy Company no.3
* Salmonella enterica *	MHI966	Agona	13	Bavaria, Germany, Dairy Company no.3

### Genome sequencing and genomic context of *lac* loci in *S. enterica* MHI strains

To understand the genetic basis of lactose metabolism and whether the *Salmonella* strains encode the canonical lactose metabolism genes *lacI* and *lacZYA*, we isolated and sequenced genomic DNA from the five strains. Combined assembly of short- and long-read DNA sequencing read data resulted in the assembly of four closed genomes (MHI916, MHI917, MHI951 and MHI966), while the final assembly of the MHI949 genome consisted of eleven contigs (Table S1). The *Salmonella* In Silico Typing Resource [SISTR, (12)] identified MHI916 and MHI917 as *Salmonella enterica* serovar Ohio (ST329) and MHI949, MHI951 and MHI966 as *Salmonella enterica* serovar Agona (ST13, Table 1). The two *S*. Ohio strains MHI916 and MHI917 had five plasmids each, while *S*. Agona MHI951 had one plasmid (Table S1). Because the genome of MHI949 was not fully assembled, we cannot report with certainty the number of plasmids in this strain.

**Fig. 2.**
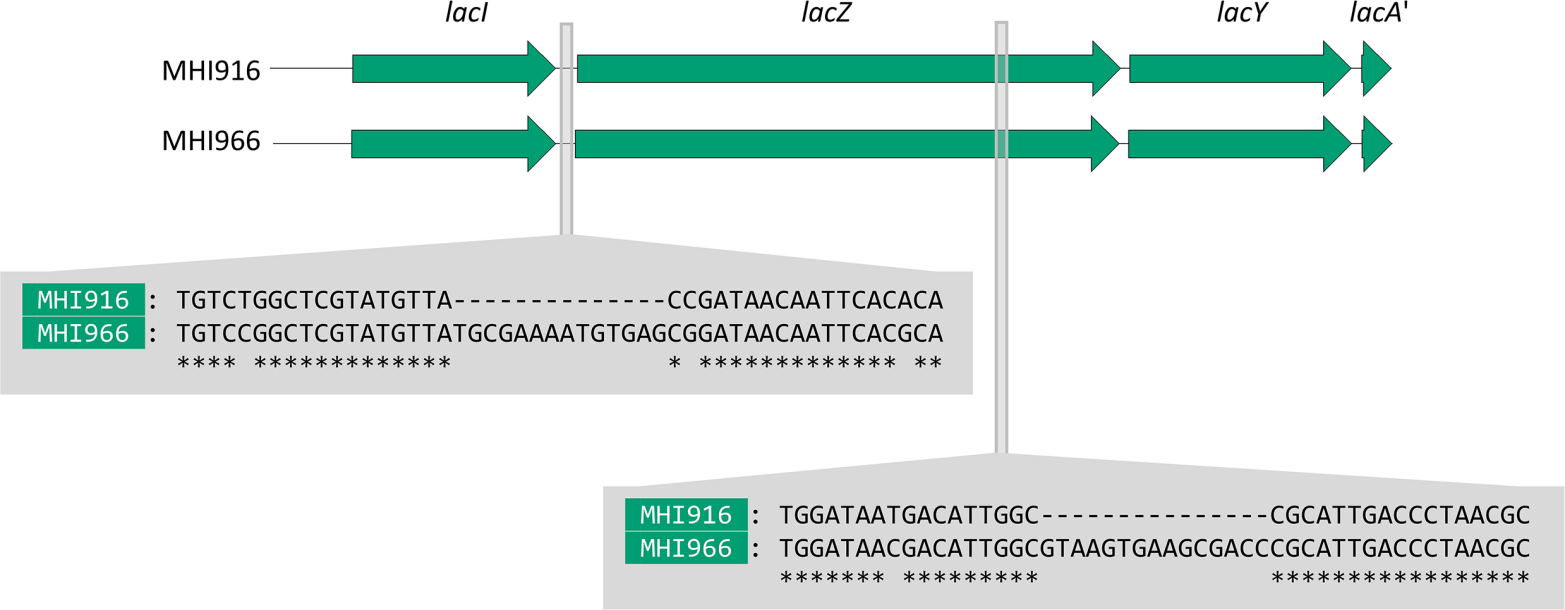
Schematic (top) and nucleotide sequence (below) alignments of the two *lac* locus variants (sequences of MHI916 and MHI966 are shown). Nucleotide sequence differences are highlighted in the grey boxes indicating the area where these differences between *lac* operons are present. Asterisks depict identical nucleotides. The *lacA* gene is truncated in all five MHI strains compared to the archetypal *E. coli lacA* gene.

Genome annotation revealed that all strains contained a *lac* locus consisting of *lacI* (transcriptional repressor LacI) and the three co-transcribed genes *lacZ* (ß-galactosidase LacZ), *lacY* (permease LacY) and *lacA* (ß-galactoside-transacetylase LacA). LacA usually has a length of 203 amino acids but was truncated in all strains to the first 47 amino acids. In the small number of *Salmonella* strains where the *lac* operon has been previously described, several are missing *lacA* [4]. Aligning the five *lac* loci revealed two sequence groups: one composed of MHI916 and MHI917 (*S*. Ohio) and a second composed of MHI949, MHI951 and MHI966 (*S*. Agona) (Fig. 2). Within each variant type, *lacI-lacZYA* sequences were identical. The two variant sequences differed in the intergenic region between *lacI* and *lacZ* and towards the latter third of the *lacZ* open reading frame (Fig. 2). Both *lacI-lacZYA* variants appear to be functional as all strains could metabolize lactose as a sole carbon source (Fig. 1).

**Fig. 3.**
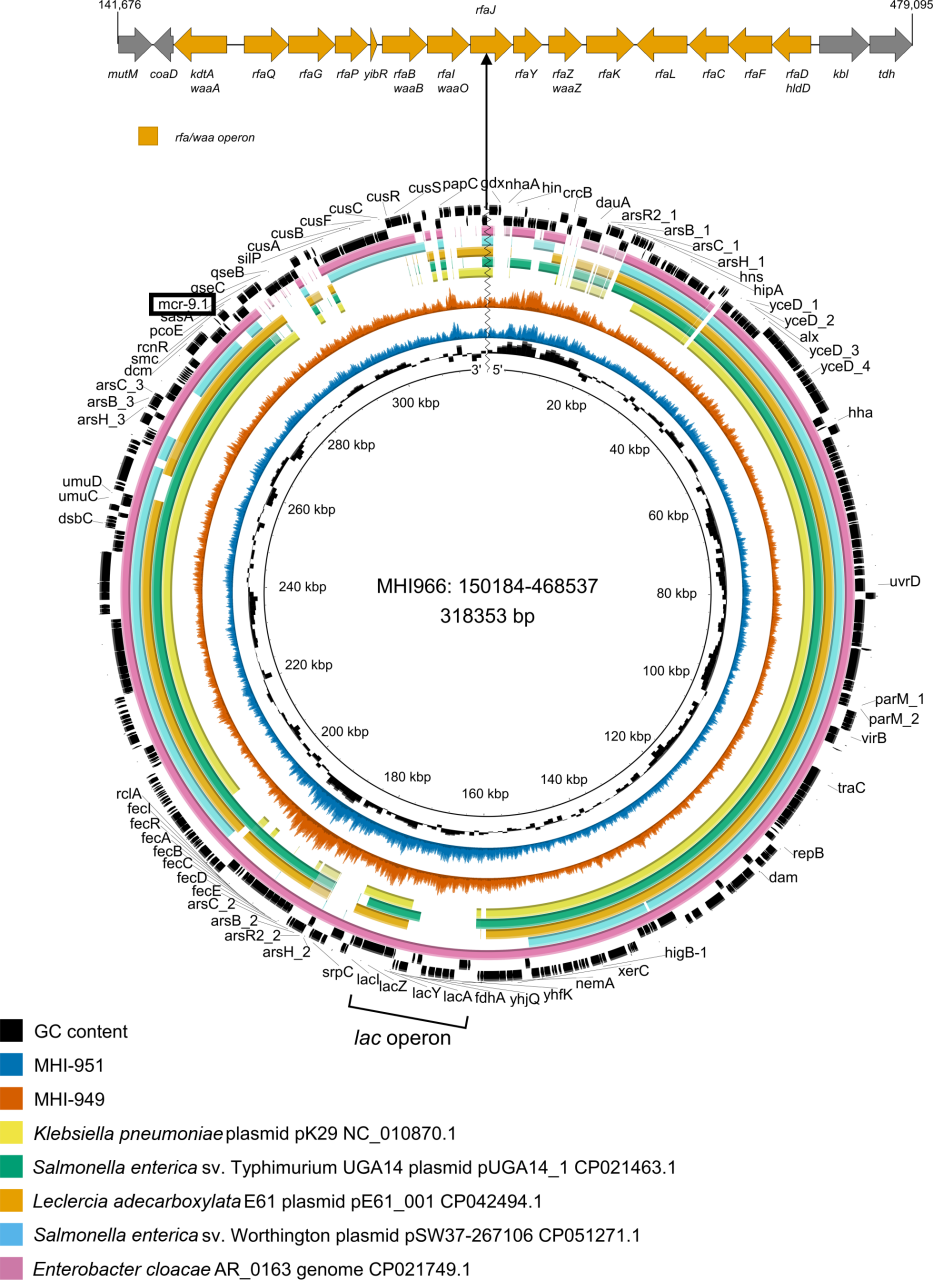
Schematic representation of the 318 kbp insertion into the *rfaJ* (*waaJ*) gene (MHI966 is depicted, top panel). The *lac* locus is highlighted with a square bracket (bottom panel). The *mcr-9* gene is located towards the top left (highlighted with a box). The two black outer rings depict genes of MHI966 with orientation of transcription. The inner, coloured rings show conservation of the 318 kbp island with closely related bacteria with similar genetic information. The orange and blue rings further in are mapped sequencing reads from MHI949 and MHI951 using MHI966 as a reference sequence. In MHI951, the island is present as a plasmid.

In *S*. Agona MHI951, the *lacI-lacZYA* locus is located in a large 318 kbp circular plasmid annotated as IncHI2A-type by MOB-suite [30]. The *lacI-lacZYA* locus was located in the same genomic context in MHI966, but in this strain the plasmidic sequence existed as a large (318,353) bp insertion inside the *waaJ* (*rfaJ*) gene (Fig. 3). In MHI949, the genomic context of *lacI-lacZYA* is the same, though we could not unequivocally determine if the plasmidic sequence was within the chromosome or a closed plasmid. The is no contiguous sequence match to the plasmid/island in GenBank, though subsections align with published sequences. The 318 kbp plasmid shows extended sequence similarity with the large plasmid pK29 found in *Klebsiella pneumoniae *, *Leclercia adecarboxylata* plasmid E61_001, *Salmonella* Worthington plasmid pSW37-267106, and an insertion into the *Enterobacter cloacae* genome (Fig. 3).

In *S*. Ohio MHI916 and MHI917, *lacI-lacZYA* was located on a 72 kbp island inserted between the genes *yqgA* and *malT* (Table 2, Fig. S1, available in the online version of this article). The island is identical between both strains, and is inserted in the genomic position where the SPI-13 island is positioned in other *S*. Typhimurium strains (e.g. 4/74 or SL1344 [38]). The 72 kbp island is an integrated plasmid as it encodes plasmidic *rep* and *tra* genes along with addiction modules characteristic of plasmids. Almost the entire island (except three genes) including *lacI-lacZYA* is conserved in two sequenced but not completely assembled lactose-positive *Salmonella* Tennessee strains: *Salmonella enterica* subsp. *enterica* serovar Tennessee strain CFSAN070643 and *Salmonella enterica* subsp. *enterica* serovar Tennessee strain CFSAN070645 (Fig. S1, [48]).

**Table 2.** Overview of genomic and phenotypic characteristics of lactose-positive *Salmonella enterica* strains

Strain	*Lac* locus	*mcr-9* locus	Motility	Colony morphology on milk agar
MHI916	Chromosome	Plasmid	Motile	Smooth, brown, precipitation area
MHI917	Chromosome	Plasmid	Non-motile	Smooth, brown, precipitation area
MHI949	Unclear	Unclear	Non-motile	Rough and dry
MHI951	Plasmid	Plasmid	Non-motile	Rough and dry
MHI966	Chromosome	Chromosome	Motile	Rough and dry

### Phylogenetic analysis of *lac* operon distribution

**Fig. 4.**
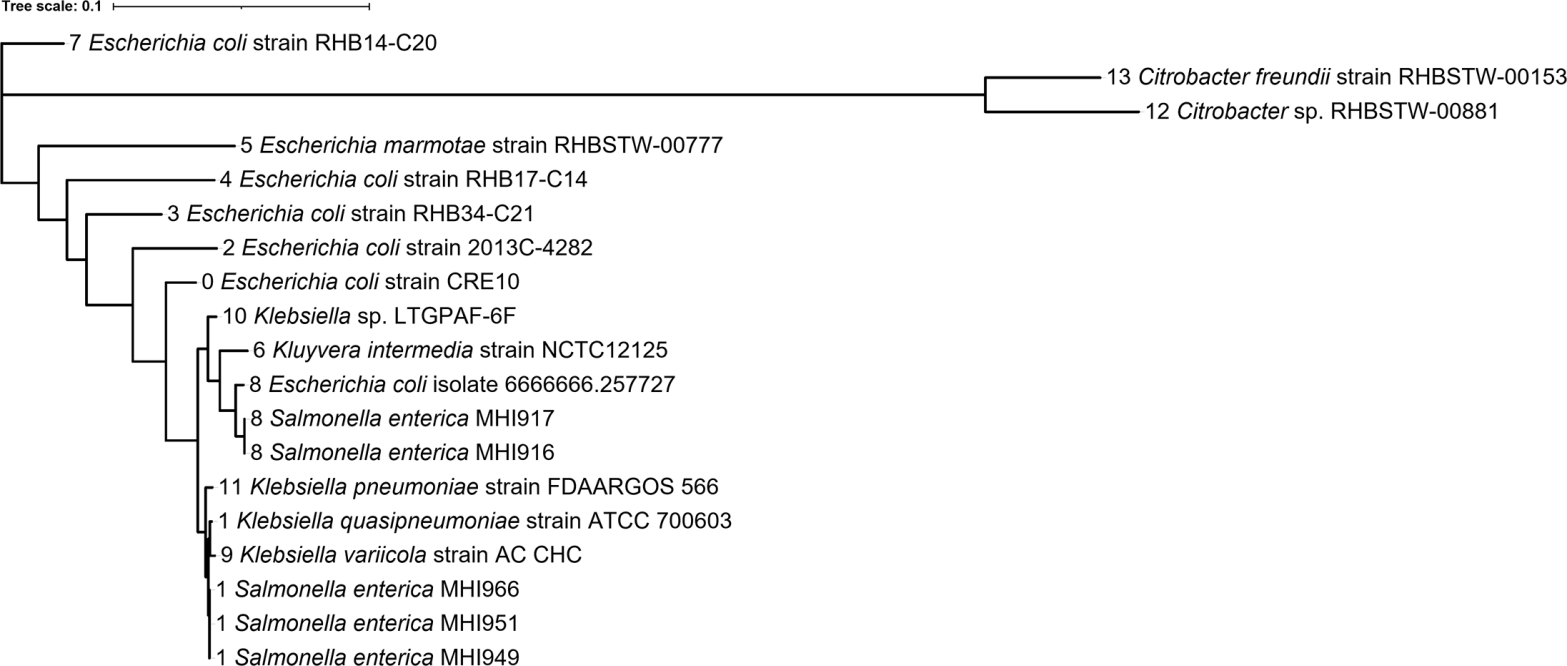
Maximum-likelihood phylogenetic tree of *lac* operon sequences. Altogether, 544 *lac* locus sequences were clustered with a 98 % nucleotide sequence identity forming 14 clusters as indicated in front of species names in the phylogenetic tree. The representatives of each cluster and *lac* locus sequences of five analysed *Salmonella enterica* genomes are shown. Strain names are given for one member of the sequence cluster. A detailed list of strains and cluster compositions can be found in .

The rarity and sporadic distribution of *lac* genes implicates horizontal gene transfer as the source of *lac* genes in the genus *Salmonella *. To examine whether the two *lacI-lacZYA* variants detected in this study are related to other mobilized *lac* genes, nucleotide sequence spanning the *lacI*, *lacZ* and *lacY* genes and intergenic regions were retrieved from the blast non-redundant nucleotide database (accessed 23 June 2021). The genome sequences were retrieved using the MHI916 *lac* locus as a query for the blastn search of loci with >90 % coverage. We obtained 539 loci representing 13 genera and the combined dataset of 544 loci (539 loci+5 *Salmonella* loci from this study) were clustered according to 98 % nucleotide sequence identity. The resulting 13 clusters are shown in Fig. 4 and . The *lac* locus of the *S*. Agona strains (MHI949, MHI951 and MHI966) was present in the by far largest cluster of sequences (*n*=382, cluster 1, Fig. 4). This *lac* locus variant is often located on plasmids, especially in *Klebsiella* species and widely distributed in the genera *Citrobacter *, *Cronobacter *, *Enterobacter *, *Escherichia *, *Klebsiella *, *Leclercia *, *Raoultella *, *Salmonella* and *Serratia* . The *S*. Ohio strains (MHI916 and MHI917) were members of the third largest cluster (*n*=31, cluster 8, Fig. 4), which were also found in *Enterobacter hormaechei *, *Cronobacter sakazakii *, *E. coli *, *Klebsiella* spp. and *Leclercia adecarboxylata* .

### Presence of an *mcr-9* gene in all five *Salmonella* MHI strains

Genome annotation revealed that all five strains contain an *mcr-9* gene (Table 2). The *mcr-9* gene variant was first described in 2019 in a multidrug-resistant *S*. Typhimurium strain [44]. In our *Salmonella* strains, four of the *mcr* genes had an identical nucleotide sequence; only the *mcr* gene from *S*. Agona MHI966 had one polymorphism (C->G) at nucleotide position 328, which was predicted to result in a Q to E amino acid change at position 110. In the *S*. Agona strains (MHI949, MHI951 and MHI966), the *mcr-9* gene was present on the same large genomic insertion/plasmid that also harboured the *lac* operon (Fig. 3). In MHI916 and MHI917, the *mcr-9* gene was also located on a large plasmid (ca. 361 k bp, IncHI1B/IncP-type) unrelated to that of the Agona strains. Thus, our data shows that the *mcr-9* gene was circulating on at least two distinct plasmids in *Salmonella enterica* populations in the 1980s.

### Antibiotic and colistin resistance

**Fig. 5.**
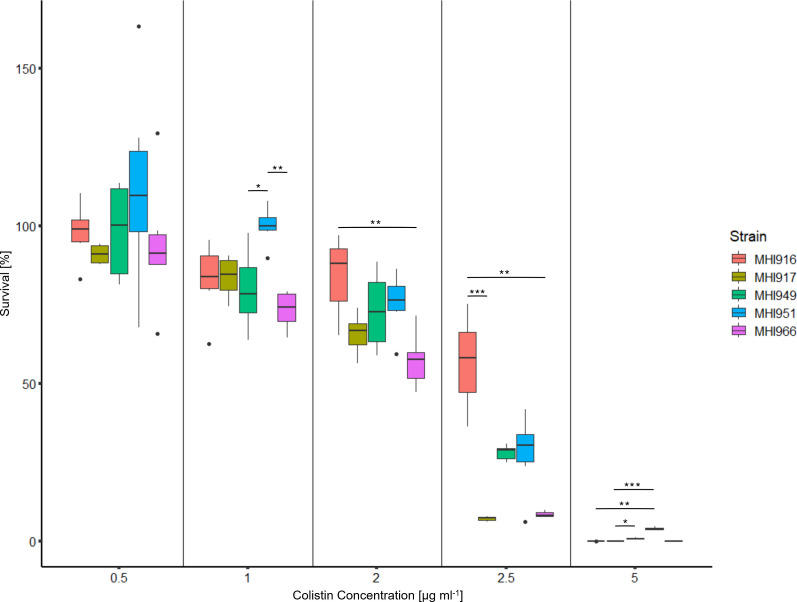
Bacteria were grown to OD_600_ 0.6–0.7 in MHI broth and colistin was added to the media at the indicated final concentrations for 1 h. c.f.u. were enumerated and % survival compared to the mock control (*n*=6). Statistical analysis was carried out with the Kruskal–Wallis test with post hoc analysis via Dunn’s multiple comparison test with statistical significance set at a *P* value of 0.05 (*P<0.05, **P<0.01, ***P<0.001).

Quantifying resistance to colistin in the lab is notoriously challenging because colistin does not diffuse well into agar [49]. Moreover, it was previously found that the presence of the *mcr-9* gene does not predict clinical levels of colistin resistance in *Salmonella enterica* serovar Typhimurium or *E. coli* [44, 50]. For example, cloning of *mcr-9* from a sensitive host conferred colistin resistance (2.5 µg ml^−1^) when ectopically expressed in a heterologous *E. coli* host, suggesting that transcriptional or translational control is critical for colistin resistance [44]. Due to the poor diffusion of colistin in regular agar, we performed the assays in soft agar to increase diffusion. Using a test strip containing a gradient of colistin concentrations (MIC test strip, 256–0.016 µg ml^−1^), two of the *Salmonella* strains (MHI916 and MHI966) were resistant to colistin (MIC at 32 and 4 µg ml^−1^), respectively . The strains MHI949 and MHI951 were equally sensitive (MIC *≤*0.016 µg ml^−1^), while MHI917 was considered sensitive (MIC 2 µg ml^−1^) but was close to the breakpoint of MIC 4 µg ml^−1^. For three strains (MHI916, MHI917 and MHI966), we observed a level of heteroresistance, as after 24 h of growth, individual colonies formed inside the inhibition zone of the colistin MIC test strips, which is not uncommon for Gram-negative bacteria [51, 52]. To further investigate the colistin resistance/sensitivity phenotypes, a broth micro-dilution (BMD) assay was carried out. We found that, in contrast to the MIC test strip assay, all *Salmonella* strains were sensitive to colistin with an MIC of <0.0625 µg ml^−1^ (the EUCAST breakpoint under the tested conditions is 2 µg ml^−1^). In addition, a colistin ‘killing assay’ was performed by adding different concentrations of colistin to exponentially growing cells in l-broth for 1 h and surviving cells were enumerated by counting colony forming units on l-agar [44]. The addition of colistin caused a concentration-dependent reduction of surviving bacteria for all the tested strains (Fig. 5). After exposure to 2 µg ml^−1^ of colistin, more than 50 % of bacterial cells were still alive for all strains; however, at 2.5 µg ml^−1^, MHI917 and MHI966 were almost completely killed, while >50 % of MHI916 cells were viable, and 25 % of MHI949 and MHI951 cells remained viable.

Except for the one polymorphism (C->G) at nucleotide position 328, no nucleotide differences were detected within or close to the *mcr-9* genes in all strains, prompting us to look more broadly for genomic differences that could account for the different levels of resistance. Comparison of the genetically very similar strains MHI916 and MHI917 found a markedly different resistance to colistin using the MIC strip and the colistin killing assay (most pronounced at 2.5 µg ml^−1^). In the more sensitive strain MHI917, a premature stop codon in the phosphoethanolamine transferase *cptA* (Fig. S4), which is regulated by *pmrA* and adds phosphoethanolamine to LPS core [53]. The CptA protein was shown to have a moderate effect on Polymxyin B resistance, but was not tested for resistance to colistin (Polymxyin E) [53]. None of the other single nucleotide variants have been associated with colistin resistance, though we did find a stop codon in MHI917 and MHI966 in the gene encoding for the alternative sigma factor σ^S^ (RpoS).

We tested for resistance to other antibiotics: penicillin G, oxacillin, cephalexin, gentamicin, trimethoprim/sulfamethoxale and ciprofloxacin. Only subtle or no differences were observed between strains (Table S2), suggesting that the differences observed with respect to colistin are not caused by general growth defects in the presence of extracellular antibiotic stressors. Genome sequence analysis with CARD [54, 55] predicted 26 antibiotic resistance genes (ARGs, Table S3) in the five *Salmonella* strains, including antimicrobial efflux pump-encoding or regulating genes *acrA*, *acrB*, *baeR*, *crp*, *ermA*, *emrB*, *emrR*, *golS*, *hns*, *kdpE*, *kpnE*, *kpnF*, *marA*, *marR*, *mdfA*, *mdtK*, *msbA*, *rsmA*, *sdiA*, *soxR*, *soxS*, *mdsA*; genes *mcr-9.1*, *pmrF*, *bacA* for alteration of antibiotic targets; and *fosA7* for antibiotic inactivation. Comparing the ARG content between the strains indicated three ARG profiles: (i) strains MHI916 and MHI917 lacked *ermA* and *fosA7*, (ii) strain MHI966 lacked the gene *marR*, and (iii) strains MHI949 and MHI951 had the complete ARG complement detected in this set of strains. The resistance genes identified by CARD can reduce sensitivities to different classes of antibiotics (monobactams, carbapenems, cephalosporins, cephamycins, penams, phenicols, penems, macrolides, fluoroquinolones, diaminopyrimidine antibiotics, aminoglycosides, aminocoumarins, tetracyclines, rifamycins, nitroimidazoles, glycylcyclines), antibiotic peptides, disinfecting agents and antiseptics.

### Production of exopolysaccharides on milk agar plates

**Fig. 6.**
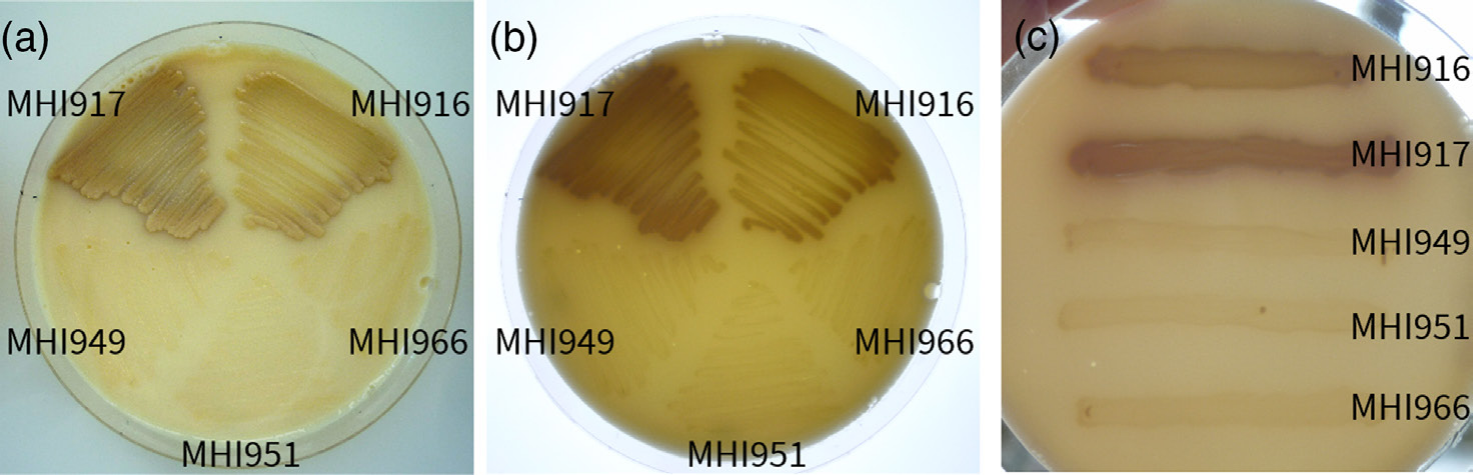
Whey-based milk agar plates showing brown colouration of *S*. Ohio MHI916 and MHI917 strains (**a–c**) and dry phenotypes of *S*. Agona MHI949, MHI951 and MHI966 (**a, c**). A brown precipitate can be seen around streak-outs of MHI916 and MHI917 (**c**). Panel (b) shows the same plate as in (a) with back-light.

Because the large genomic insertion in *S. enterica* MHI966 was present in the gene *rfaJ* (*waaJ*), which encodes an α−1,2-glucosyltransferase in *E. coli* and *Salmonella* [56–58], we investigated whether this insertion influences colony morphology, because reduced lipopolysaccharide production can lead to a dry colony phenotype. The protein RfaJ adds an α −1,2-glucose to the O antigen of the lipopolysaccharide in *Salmonella* [56]. When all five strains were streaked out on whey-based formula milk agar and incubated for 24 h at 37 °C and then 4 days at room temperature, we noted that strain MHI966, but surprisingly also MHI949 and MHI951, produced colonies that were markedly less mucoid than MHI916 and MHI917, indicating that less exopolysaccharide was produced, likely caused by the disruption of *rfaJ* in MHI966. In investigating the genomes of MHI949 and MHI951, we discovered that they had a frameshift mutation in the *rfaL* gene, which encodes an O-antigen ligase; this frameshift was absent in MHI966 and could potentially explain the dry phenotype of MHI949 and MHI951 on milk agar. The strains MHI916 and MHI917 also showed a light brown colouring and were surrounded by a brown zone of precipitation, while the other three strains remained beige/creamy and dry (Fig. 6, Table 2).

### Heavy-metal resistance

Heavy metals are widely used in animal feed to stimulate growth and act as antimicrobial agents. In the 1980s, heavy metals and antibiotics were used extensively to prevent animal disease, which could explain why antibiotic and heavy-metal resistance genes are often co-selected [7]. All five *Salmonella* strains had genes for resistance or tolerance to arsenic (*ars*), copper (*cus*, *copA*, *pcoE*, *cutC*, *cueO*), tellurite (*ter*, *tehAB*), silver (*silE*, *silP*), gold (*golS*) and cadmium (*zntA*). Two separate arsenic resistance cassettes *arsRBCH* and *arsRDCAB* occurred in two to three copies depending on the strain and plasmids. Genes encoding tellurite, copper and arsenic resistance were located on plasmids as well as on the chromosomes of all *Salmonella* strains. In contrast, cadmium and gold resistances were encoded only on the chromosome and silver resistance only on plasmids.

The genomic analysis revealed that many heavy-metal resistance or tolerance genes were located in a genomic island called *Salmonella* genomic island 4 (SGI4), which is located in all isolates on plasmids, except for MHI966, where the island is part of the large chromosomal insertion that also contains the *lac* locus [59–61] (Fig. 3). SGI-4 consists of several open reading frames, including a copper homeostasis and silver resistance island (CHASRI), an arsenic tolerance operon and diverse transposable genetic elements, e.g. insertion sequences (IS). Usually, the CHASRI consists of a copper homeostasis system (*pco*) cluster (*pcoABCDRSE*), a *sil* heavy-metal export system cluster (*silE*, *silP*), and a copper-sensing copper efflux system (*cus*) cluster (*cusABFCRS*) [59]. A particularly notable feature of MHI949, MHI951 and MHI966 was the insertion of *mcr-9*, *wbuC* and *qseCB* genes, flanked by IS elements and inserted between *pcoE* and *silP* in the CHASRI.

### Motility of MHI strains

Typically, *Salmonella* bacteria possess flagella that propel them in liquid media. Because flagella are important for *Salmonella* pathogenicity, the swimming behaviours of the lactose-fermenting *Salmonella* strains in the presence of different amounts of lactose were evaluated using a soft agar motility assay [three different media l-agar, L-agar +lactose, XLD +lactose (XLDmod)], (Fig. S2,Table 2). We used a modified XLD in addition to l-agar, because it is one of the selective media used to detect *Salmonella* in food samples. When *Salmonella* strains were stab-inoculated into three different media, only strains *S*. Typhimurium 4/74, MHI916 and MHI966 showed any motility (motility zone of ca. 2–4.5 cm) after incubation for 8 h at 37 °C (Fig. S2). In contrast, MHI917, MHI949 and MHI951 were non-motile and only grew at the site of stab inoculation (Fig. S2). *Salmonella* strains MHI917, MHI949 and MHI951 remained non-motile even after 24 h incubation at 37 °C on LB with lactose and XLDmod; however, after 24 h of incubation on LB, MHI949 and MHI951 showed weak motility (motility zone diameter 1.3–2.2 cm), indicating that the presence of lactose in the medium might have a mild detrimental effect on motility.

The genome sequences were searched for mutations in genes involved in motility. The nonmotile strain MHI917 had a single nucleotide polymorphism in the *flgF* gene causing a stop codon at amino acid position 106 (Fig. S3). FlgF is a proximal subunit of the flagellar rod and an early-type secretion substrate of the flagellar secretion system in *S*. Typhimurium [62, 63]. In *E. coli *, mutation of *flgF* resulted in a non-motile phenotype [64]; therefore, we predict that the stop codon in *flgF* is responsible for the motility defect observed in MHI917. For strains MHI949 and MHI951, we could not pinpoint the mutations that are responsible for their lack of motility.

## Discussion

Lactose fermentation is rare in *Salmonella* isolates. A study in 1973 revealed that 86 out of 552(15.6 %) of *Salmonella* strains (31 different serovars) isolated from 175 dried milk and milk-drying plants in the US were lactose fermenting [65]. Presence of the *lac* operon in *S. enterica* was demonstrated in 2015 by genome sequencing and phylogenetic analyses [4]. In the present study, five isolates from powdered milk in Germany in the 1980s were experimentally validated to use lactose as a sole carbon source, which is advantageous in a dairy-production setting due to the high abundance of lactose in milk and milk powder. Whole-genome sequencing identified serovars Ohio and Agona encoding two variant *lac* operons, each with a truncated *lacA*. In *Salmonella *, LacA represses flagella expression but does not contribute to lactose metabolism [66], thus this dispensable component of the *lac* operon may be counter-selected due to interference with motility.

Our findings also reveal that two variants of *mcr-9* were present in two distinct geographical locations in Germany in the 1980s. All five strains possessed a copy of an *mcr-9* gene and in at least three of the five strains, the *mcr-9* gene is located on a plasmid. Colistin has been used continuously in veterinary medicine since the 1950s, mostly to treat gastrointestinal infections with Gram-negative bacteria and more recently as a last resort drug to treat infections by *Enterococcus faecium *, *Staphylococcus aureus *, *Klebsiella pneumoniae *, *Acinetobacter baumannii *, *Pseudomonas aeruginosa* and *Enterobacter* spp. (ESKAPE) pathogens in humans [67, 68]. The prototypical mobile colistin resistance gene (*mcr-1*) was discovered in China in 2015, making headlines in global media [69]. The *mcr-1* gene was subsequently found in *E. coli* isolated from chickens in China in the 1980s and a *Salmonella* strain from 2007 [70, 71]. Ten *mcr* gene variants have now been described [72], and a recent study suggests the origin of *mcr-9* in *Salmonella enterica* Germany is consistent with our data [44, 73].

Our results highlight the problematic nature of measuring colistin resistance in the lab, consistent with several studies that found that the presence of *mcr-9* gene is not sufficient to confer colistin resistance [50, 74, 75]. Nevertheless, over-expression of *mcr-9* consistently generates clinically relevant resistance in *E. coli* [44]. We used three different assays (MIC test strips, BMD and a colistin killing assay) to evaluate the level of colistin resistance and all have their limitations. In the gold standard micro-broth dilution assay, none of our five strains showed a clinically relevant level of resistance showing that the presence of *mcr-9* is a poor predictor of resistance in this assay. However, using MIC strips and a colistin killing assay [44], the picture became more complex. Colistin resistance was reproducibly higher in MHI916 than MHI917 despite identical *mcr-9* genes and genomic contexts suggesting that low-level colistin resistance is multi-factorial and not solely mediated by the presence of *mcr-9*. QseBC is implicated to contribute to regulation of *mcr-9* [76], but the *qseBC* genes were also identical in the two strains. Deletion of *cptA* has been noted to cause higher sensitivity to polymyxin B [53], and this gene has a premature stop codon in MHI917. More mechanistic work and a detailed genetic dissection, for example, gene deletions and overexpression studies, are required to understand the regulation of *mcr-9* and *cptA* gene expression, function and interplay with other genes to confer colistin resistance.

Cattle are exposed to heavy metals through multiple sources. Heavy metals are used as animal feed additives for their growth-stimulating properties (arsenic, copper, zinc and nickel), for prevention of microbial contamination in wounds (silver, copper) [77–79]. Heavy metals (arsenic, copper) are used as different biocides like herbicides, insecticides and fungicides, where they were used as plant growth regulators (arsenic) or fertilisers (cadmium, copper), and can be general feed contaminants (mercury, cadmium, tellurium) [77–79]. Industrialization, agriculture, and human and veterinary medicine have led to further widespread environmental contamination by heavy metals [80]. Pathogenic bacteria also encounter heavy metals as a host defence mechanism against invading microorganisms. To cope with heavy metal toxicity, micro-organisms have evolved different detoxification mechanisms like reduction of heavy metals or their efflux [81]. Arsenic, cadmium, copper, mercury, silver, tellurium and zinc, for instance, have shown antimicrobial activity due to their involvement in production of reactive oxygen species (ROS), protein dysfunction and loss of enzymatic activity, membrane potential disturbance and more [79].

In the five *Salmonella* isolates, numerous detoxification systems were predicted, including arsenic detoxification genes. The main sources of arsenic contamination on dairy farms are animal feed supplementation for growth-stimulation and indiscriminate use of arsenic pesticides [81]. In our *Salmonella* isolates, we found two types of arsenic resistance determinants *arsRBCH* and *arsRDCAB*, that confer low-level and high-level arsenic resistance, respectively, in *E. coli* [82, 83]. The presence of mobile genetic elements, such as plasmids, suggest acquisition of arsenic resistance clusters via horizontal gene transfer and additionally has been found to promote faster dissemination of ARGs [84]. Studies have shown that the supplementation of cattle feed with copper at higher than the physiological requirements are correlated with an increased prevalence of copper-resistant faecal *Enterococci* and a co-selection for macrolide resistance, which can been identified also in agricultural soils [80]. Multiple mechanisms confer copper resistance, including copper homeostasis (*copA*, *copB* and *cutC*) or copper efflux (*cusABFCRS*); both were present in our *Salmonella* isolates. The plasmid-borne *cus*-cluster we observed in SGI-4 was previously associated with *Salmonella* copper tolerance under anaerobic conditions suggesting that it might play a role in the mammalian intestinal tract [59]. The *sil*-genes (*silEP*) encode the SilE periplasmic silver binding-protein and SilP silver efflux pump. In veterinary medicine, silver has been used for the treatment of wounds and has been applied for water treatment [85].

The *Salmonella* strains showed additional phenotypic diversity that correlated with specific genotypes. The strains MHI949, MHI951 and MHI966 showed a dry phenotype on milk agar plates, which could be explained by loss of function in LPS biosynthesis due to frame shift mutations in *rfaL* in MHI949 and MHI951 and the insertion of the large 318 kbp plasmidic island into the LPS biosynthesis (*rfa*/*waa*) gene cluster in MHI966. Three strains (MHI917, MHI949 and MHI951) were non-motile. We discovered the mutation that could explain the MHI9147 phenotype: MHI917 possessed a stop codon in the gene encoding for the rod protein FlgF, while we could not find the mutations for the lack of motility of MHI949 and MHI951. Other notable mutations were premature stop codons in the *rpoS* gene in MHI917 and MHI966, but it is unclear whether RpoS plays a role in any of the observed phenotypes. Although reduced motility and RpoS regulation are expected to impair virulence, reduced invasiveness might be offset by the ability to use lactose as a carbon source and by the acquisition of metal detoxification genes, both of which can support growth in cattle intestines and in industrial-scale milk production.

## Supplementary Data

Supplementary material 1

Supplementary material 2
