# Supplementary material for: Plasmid-encoded lactose metabolism and mobilized colistin resistance (mcr-9) genes in Salmonella enterica serovars isolated from dairy facilities in the 1980s
Source: Microb Genom. 2023 Nov 30;9(11):001149. doi: 10.1099/mgen.0.001149 (PMC10711319; doi:10.1099/mgen.0.001149)
Supplement: Supplementary material 1 [file mgen-9-1149-s001.pdf]

Plasmid-encoded lactose metabolism and mobilised colistin resistance (*mcr-9*) genes in *Salmonella enterica* serovars isolated from dairy facilities in the 1980s.

Supplementary Material

Kröger *et al*.

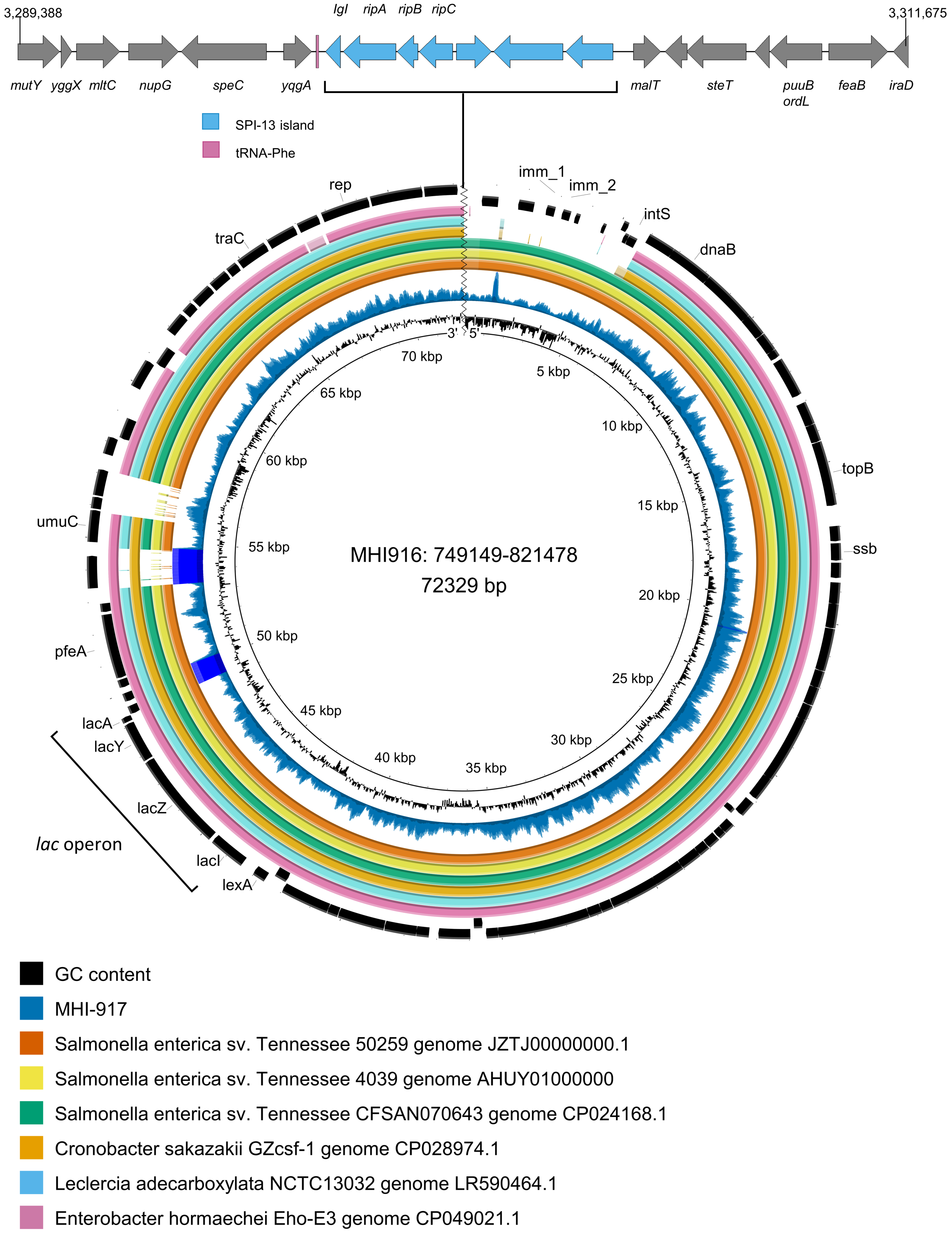

Supplementary Figure 1: The *lac* locus of MHI916 is highlighted with a square bracket. The two black outer rings depict genes of MHI966 with orientation of transcription. The inner, coloured rings show conservation of the 72.3 kbp island with closely related bacteria with similar genetic information.

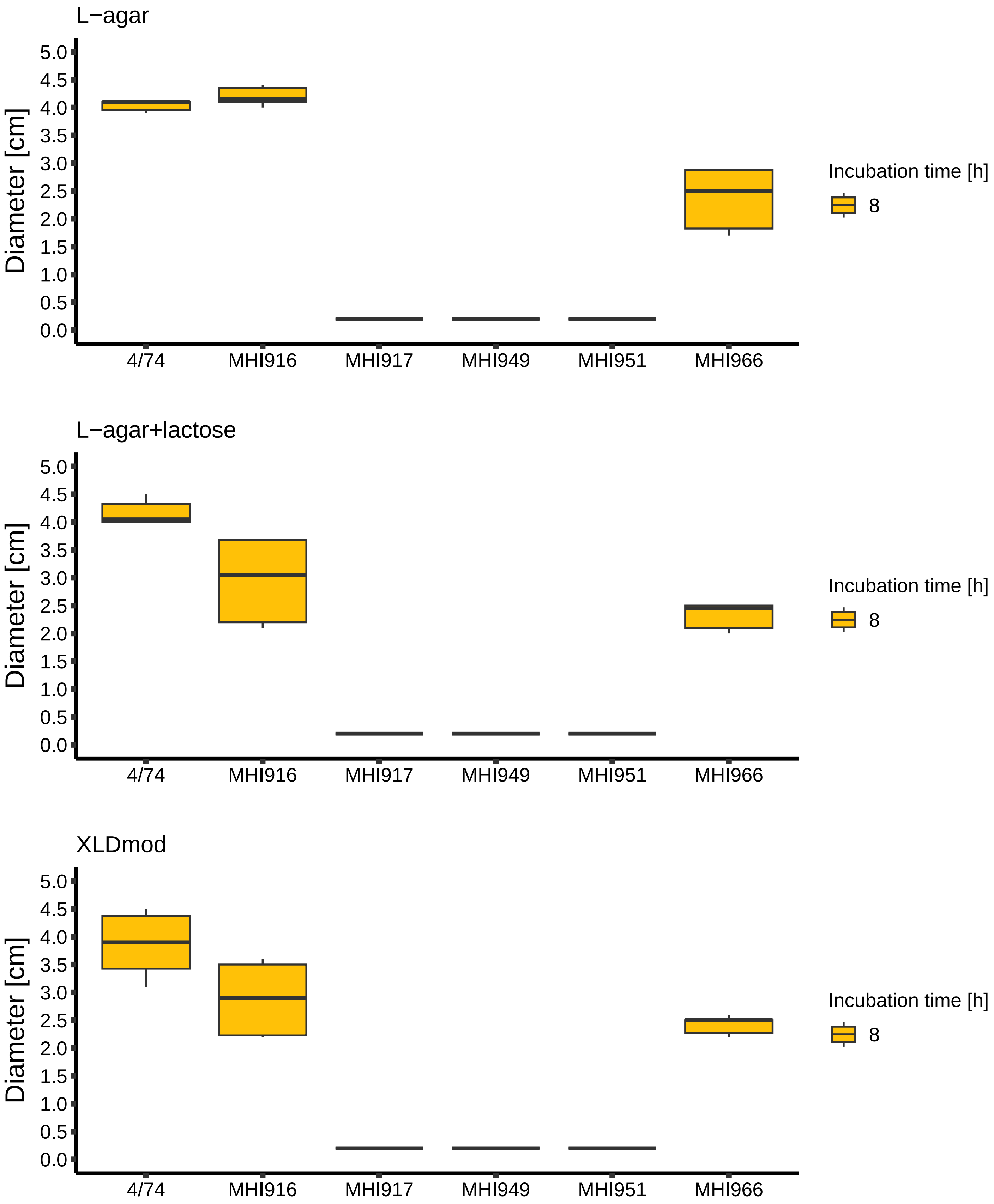

Supplementary Figure 2: Motility of strains. The indicated bacterial strains were stab-inoculated into L-agar (top panel), L-agar+lactose (middle panel) and XLDmod (lower panel) soft agar and incubated for 8 hours at 37°C before their motility zone was measured. The strains *S*. Typhimurium 4/74, MHI916 and MHI966 showed the ability to swim within the soft agar, while MHI917, MHI949 and MHI951 did not display any motility after 8 hours of incubation (n=6).

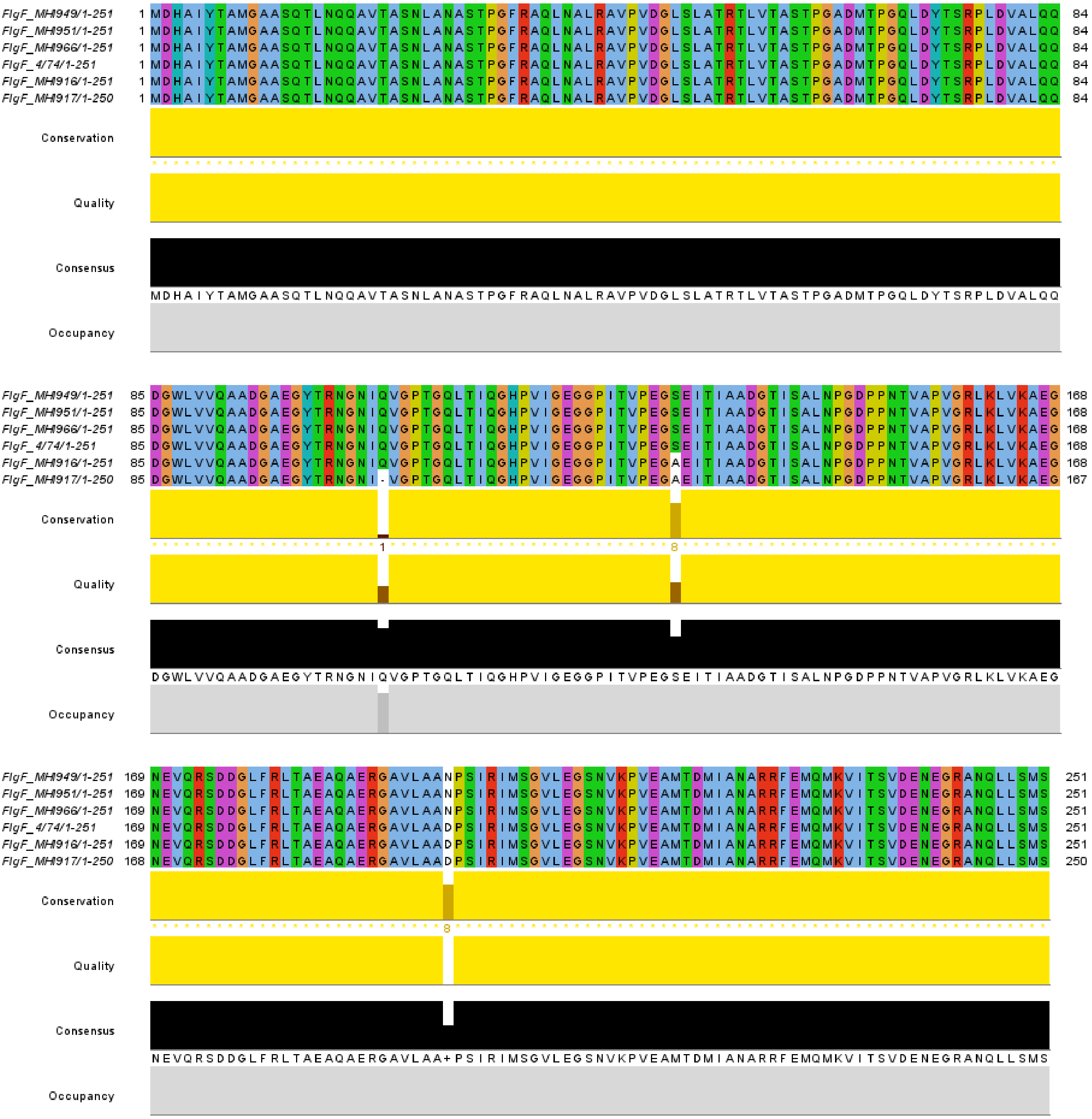

Supplementary Figure 3: Multi sequence alignment of FlgF protein sequences using Clustal Omega and visualised with Jalview revealing the presence of a stop codon at position 106 in strain MHI917, which could be responsible for the non-motile phenotype of MHI917.

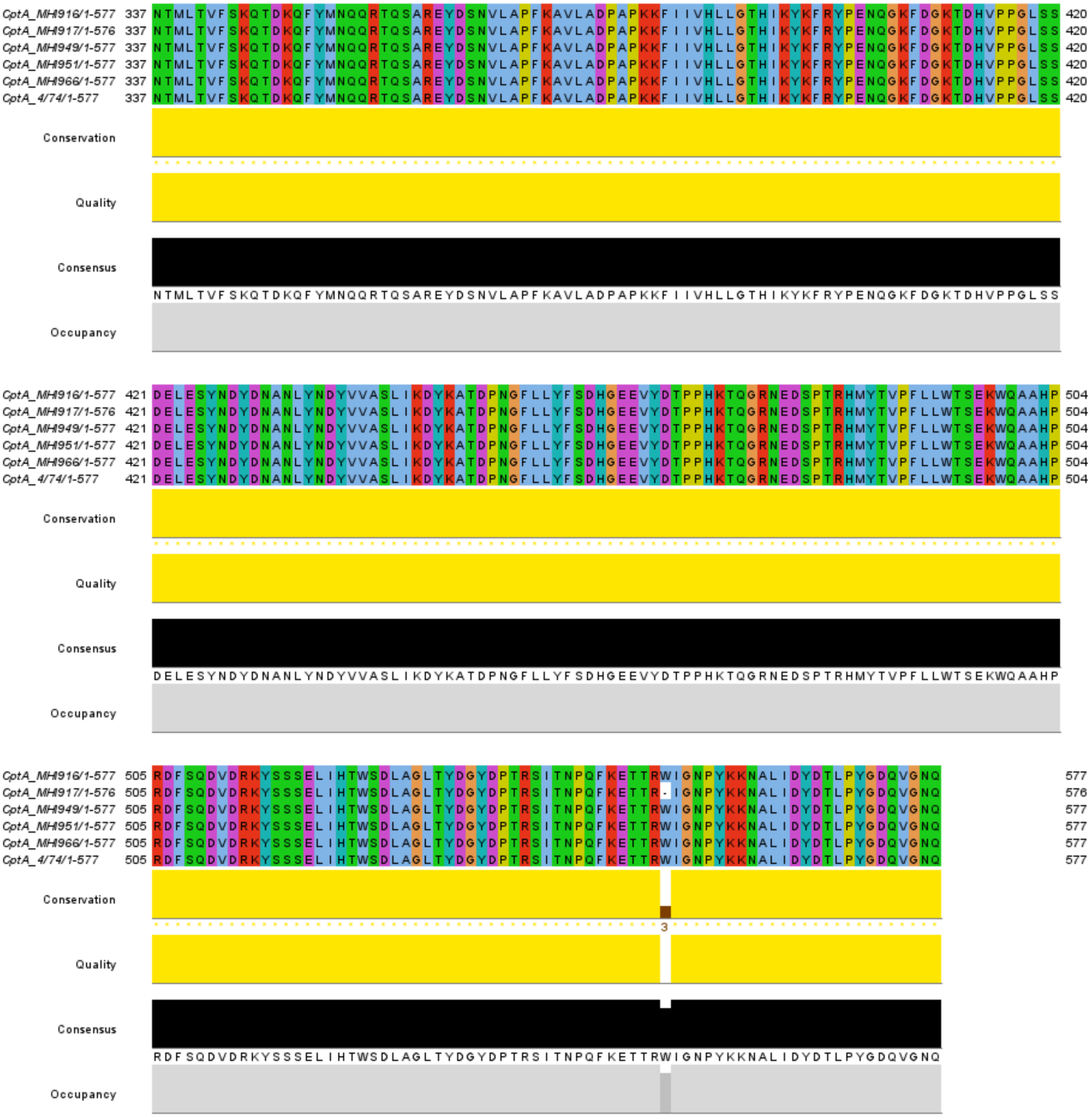

Supplementary Figure 4: Multi sequence alignment of CptA protein (577 aa) sequences using Clustal Omega and visualised with Jalview revealing the presence of a stop codon at position 552 in strain MHI917, which could be responsible for the lower colistin resistance of MHI917. The alignment is shown for amino acids 337-577. No amino acids exchanges were detected between aa 1-336.

Supplementary Table 1. Sequencing and genome statistics of lactose-positive *Salmonella enterica* strains.

Species	Strain	Genome size [bp]	GC [%]	Plasmids	Genome sequencing status
Salmonella enterica	MHI916	5,258,195	52.2	5	Closed
Salmonella enterica	MHI917	5,258,746	52.2	5	Closed
Salmonella enterica	MHI949	5,140,919	51.8	unclear	11 contigs
Salmonella enterica	MHI951	5,176,702	51.8	1	Closed
Salmonella enterica	MHI966	5,130,882	51.8	0	Closed

Supplementary Table 2. Antibiotic resistance of *Salmonella enterica* strains determined by disk diffusion (penicillin G [10 µg], oxacillin [5 µg], cephalexin [30 µg], gentamicin [10 µg], trimethoprim/sulfamethoxazole (TMP/SMX, 25 µg), ciprofloxacin (5 µg); zone diameter in mm) or MIC test strip (colistin only). Sensitivity (S), Intermediate (I) or Resistance (R) are assigned according to EUCAST breakpoints (CSLI standard for MIC test strip).

Strain	Penicillin G [mm]	Oxacillin [mm]	Cephalexin [mm]	Gentamicin [mm]	TMP/SMX [mm]	Ciprofloxacin [mm]	Colistin [µg/mL]
MHI916	0 (R)	0 (R)	19 (S)	19 (S)	29 (S)	28 (S, I)	32 (R)
MHI917	0 (R)	0 (R)	19 (S)	19 (S)	30 (S)	28 (S, I)	2 (S)
MHI949	0 (R)	0 (R)	21 (S)	19 (S)	31 (S)	29 (S, I)	≤0.016 (S)
MHI951	0 (R)	0 (R)	21 (S)	19 (S)	33 (S)	30 (S, I)	≤0.016 (S)
MHI966	0 (R)	0 (R)	20 (S)	20 (S)	32 (S)	30 (S, I)	4 (R)

Supplementary Table 3. Antimicrobial resistance gene (ARG) profiles detected among the lactose fermenting Salmonella enterica strains predicted by CARD.

Strain	Target Alteration	Efflux	Inactivation
MHI916	*mcr-9.1, pmrF, bacA*	*acrA, acrB, baeR, crp, emrB, emrR, golS, hns, kdpE, kpnE, kpnF, marA, marR, mdfA, mdtK, msbA, rsmA, sdiA, soxR, soxS, mdsA*	-
MHI917	*mcr-9.1, pmrF, bacA*	*acrA, acrB, baeR, crp, emrB, emrR, golS, hns, kdpE, kpnE, kpnF, marA, marR, mdfA, mdtK, msbA, rsmA, sdiA, soxR, soxS, mdsA*	-
MHI949	*mcr-9.1, pmrF, bacA*	*acrA, acrB, baeR, crp, ermA, emrB, emrR, golS, hns, kdpE, kpnE, kpnF, marA, marR, mdfA, mdtK, msbA, rsmA, sdiA, soxR, soxS, mdsA*	fosA7
MHI951	*mcr-9.1, pmrF, bacA*	*acrA, acrB, baeR, crp, ermA, emrB, emrR, golS, hns, kdpE, kpnE, kpnF, marA, marR, mdfA, mdtK, msbA, rsmA, sdiA, soxR, soxS, mdsA*	fosA7
MHI966	*mcr-9.1, pmrF, bacA*	*acrA, acrB, baeR, crp, ermA, emrB, emrR, golS, hns, kdpE, kpnE, kpnF, marA, mdfA, mdtK, msbA, rsmA, sdiA, soxR, soxS, mdsA*	fosA7
